# Supplementary material for: Identification of photoperiod-induced specific miRNAs in the adrenal glands of Sunite sheep (Ovis aries)
Source: Front Vet Sci. 2022 Jul 22;9:888207. doi: 10.3389/fvets.2022.888207 (PMC9354845; doi:10.3389/fvets.2022.888207)
Supplement: Supplementary file 1 [file Data_Sheet_1.ZIP › Supplementary file/miRDeep2 file.zip!/miRDeep2 file/Know_Structure/SPLP42A2/oar-mir-22.pdf]

miRBase precursor : oar-mir-22
Total read count : 109107
oar-miR-22-3p read count : 102604
remaining reads : 6503

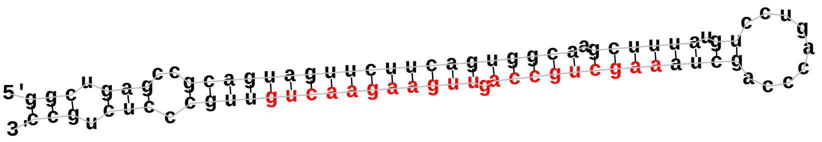

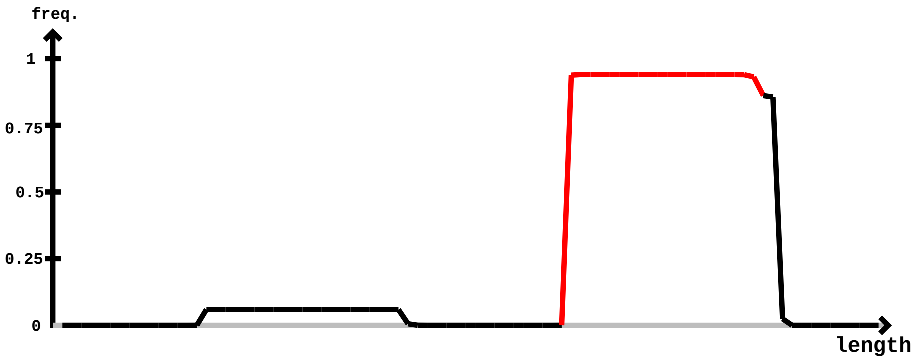


oar-miR-22-3p

```
5'- ggcugagccgcaguaguucuucaguggcaagcuuuauguccugacccagcuaaagcugccaguugaagaacuguugcccucugcc   -3'    exp
    (((.(((..((((((((((((((((((((.((((((.((.........))))))))))))).))))))))))))))).))).)))    reads    mm    sample
    .............uaguucuucaguggcaagcuuu..................................................    15       0     seq
    .............uaguucuucaguggcaagcuuua.................................................    1        0     seq
    ..............aguucuucaguggcaagc.....................................................    2        0     seq
    ..............aguucuucaguggcaagcu....................................................    3        0     seq
    ..............aguucuucaguggcaagcuu...................................................    56       0     seq
    ..............aguucuucaguggcaagcuuu..................................................    5794     0     seq
    ..............aguucuucaguggcaagcuuua.................................................    590      0     seq
    ..............aguucuucaguggcaagcuuuau................................................    32       0     seq
    ...............guucuucaguggcaagcuuu..................................................    9        0     seq
    ................uucuucaguggcaagcuuuaugu..............................................    1        0     seq
    ...................................................aaagcugccaguugaagaac..............    1        0     seq
    ...................................................aaagcugccaguugaagaacu.............    7        0     seq
    ...................................................aaagcugccaguugaagaacugu...........    14       0     seq
    ....................................................aagcugUcaguugaaga................    1        1     seq
    ....................................................aagcugccaguugaaga................    17       0     seq
    ....................................................aagcugccaguugaagaa...............    45       0     seq
    ....................................................aagcugccaguugaagaac..............    903      0     seq
    ....................................................aagcugccaguugaagaacu.............    7627     0     seq
    ....................................................aagcugccaguugaagaacug............    592      0     seq
    ....................................................aagcugccaguugaagaacugu...........    90506    0     seq
    ....................................................aagcugccaguugaagaacuguu..........    2635     0     seq
    ....................................................aagcugccaguugaagaacuguug.........    4        0     seq
    .....................................................agcugccaguugaagaac..............    3        0     seq
    .....................................................agcugccaguugaagaacu.............    14       0     seq
    .....................................................agcugccaguugaagaacugu...........    210      0     seq
    .....................................................agcugccaguugaagaacuguu..........    22       0     seq
    ......................................................gcugccaguugaagaacuguu..........    3        0     seq
```
